# Validation of prediction models: examining temporal and geographic stability of baseline risk and estimated covariate effects

**DOI:** 10.1186/s41512-017-0012-3

**Published:** 2017-04-13

**Authors:** Peter C. Austin, David van Klaveren, Yvonne Vergouwe, Daan Nieboer, Douglas S. Lee, Ewout W. Steyerberg

**Affiliations:** 1 Institute for Clinical Evaluative Sciences, G106, 2075 Bayview Avenue, Toronto, Ontario M4N 3M5 Canada; 2 Institute of Health Policy, Management and Evaluation, University of Toronto, Toronto, Canada; 3 Schulich Heart Research Program, Sunnybrook Research Institute, Toronto, Canada; 4 Department of Public Health, Erasmus MC-University Medical Center Rotterdam, Rotterdam, The Netherlands; 5 Predictive Analytics and Comparative Effectiveness Center, Institute for Clinical Research and Health Policy Studies, Tufts Medical Center, Boston, MA USA; 6 Department of Medical Statistics and Bioinformatics, Leiden University Medical Center, Leiden, The Netherlands; 7 Peter Munk Cardiac Centre and Joint Department of Medical Imaging, Division of Cardiology, Department of Medicine, University of Toronto, Toronto, Canada

**Keywords:** Clinical prediction model, Validation, Risk prediction, Hierarchical regression model, Geographic variation, Temporal variation

## Abstract

**Background:**

Stability in baseline risk and estimated predictor effects both geographically and temporally is a desirable property of clinical prediction models. However, this issue has received little attention in the methodological literature. Our objective was to examine methods for assessing temporal and geographic heterogeneity in baseline risk and predictor effects in prediction models.

**Methods:**

We studied 14,857 patients hospitalized with heart failure at 90 hospitals in Ontario, Canada, in two time periods. We focussed on geographic and temporal variation in baseline risk (intercept) and predictor effects (regression coefficients) of the EFFECT-HF mortality model for predicting 1-year mortality in patients hospitalized for heart failure. We used random effects logistic regression models for the 14,857 patients.

**Results:**

The baseline risk of mortality displayed moderate geographic variation, with the hospital-specific probability of 1-year mortality for a reference patient lying between 0.168 and 0.290 for 95% of hospitals. Furthermore, the odds of death were 11% lower in the second period than in the first period. However, we found minimal geographic or temporal variation in predictor effects. Among 11 tests of differences in time for predictor variables, only one had a modestly significant *P* value (0.03).

**Conclusions:**

This study illustrates how temporal and geographic heterogeneity of prediction models can be assessed in settings with a large sample of patients from a large number of centers at different time periods.

## Background

Clinical prediction models permit one to estimate the probability of the presence of disease (diagnosis) or the probability of the occurrence of adverse events for patients with specific medical diagnoses or undergoing specific surgical procedures or interventions (prognosis). Classical aspects of model validation include internal validation or reproducibility (how the model performs in patients who were not included in model development, but who are from the same underlying population), temporal validation (how the model performs on subsequent patients at the same centers at which the model was developed), and geographic validation (how the model performs on patients from centers different from those which participated in model development) [1–5]. The current gold standard approach to assessing model validity is to report a summary measure of model performance, such as the concordance statistic (*c*) or area under the ROC curve, in a sample different from that in which the model was developed [6]. We have previously illustrated methods for assessing the temporal and geographic performance of prediction models in independent samples [7]. In the current study, we describe how to examine the stability of a model’s baseline risk and predictor effects across time and geography.

Model transportability can also be examined by the temporal or geographic stability of the baseline risk and predictor effects. A desirable property for a prediction model is that the estimated effects are constant across geographic regions and across different temporal periods. Our objective was to describe and illustrate methods for assessing such geographic and temporal stability of baseline risk and predictor effects and to provide guidance on their use. Accordingly, we analyzed data on patients hospitalized with congestive heart failure (CHF) at 90 hospitals in two time periods.

## Methods

### Data source and prediction model

The current study used 7549 patients hospitalized with CHF during the first phase of the EFFECT study phase (April 1999 to March 2001) and 7308 patients hospitalized with CHF during the second phase of the study (April 2004 to March 2005) [8]. Trained cardiovascular nurse abstractors retrospectively abstracted data on patient demographics, vital signs and physical examination at presentation, medical history, and results of laboratory tests from patients’ medical records. The abstracted data were linked to the Registered Persons Database for determination of the vital status of each subject. These data were linked using unique, encoded identifiers and were analyzed at the Institute for Clinical Evaluative Sciences.

The EFFECT-HF mortality prediction model for 1-year mortality uses 11 variables: age, systolic blood pressure on admission, respiratory rate on admission, low-sodium serum concentration (<136 mEq/L), low serum hemoglobin (<10.0 g/dL), serum urea nitrogen, presence of cerebrovascular disease, presence of dementia, chronic obstructive pulmonary disease, hepatic cirrhosis, and cancer [9]. For the current analyses, the four continuous variables were centered to have mean zero. This was done so that the model intercept would be interpretable as pertaining to a person with none of the binary (yes/no) risk factors and who is average on all the continuous factors. Greater details on the study sample and prediction model are provided elsewhere [7, 9]. All analyses were conducted in the pooled sample consisting of patients from both phases of the study.

### Exploring geographic heterogeneity

First, we fit a fixed effects logistic regression model in which the probability of 1-year mortality was regressed on the 11 predictors in the EFFECT-HF model (model 1) (all models are described mathematically in the Appendix). This model ignores both temporal and geographic variability in the probability of 1-year mortality. From this model, we extracted the fitted linear predictor. This is the conventional linear predictor that would be obtained in a study that ignored temporal and geographic effects. This linear predictor will be used in subsequent models where noted.

A series of random effects logistic regression models were fit to explore geographic variation. First, we modified Model 1 by including hospital-specific random intercepts (Model 2). The inclusion of random intercepts allows one to explore geographic variation in the baseline risk of 1-year mortality across hospitals, by allowing the log-odds of 1-year mortality to vary across hospitals. While the intercept was allowed to vary across hospitals, the effect of each predictor variable was assumed to be constant across hospitals.

We then fit a random intercept model in which the log-odds of 1-year mortality was regressed on the marginal linear predictor estimated from Model 1 (we refer to this new model as Model 3). This analysis allows one to assess whether the log-odds of death for an arbitrarily-defined reference patient (one whose linear predictor was equal to zero) varies across hospitals. As above, the effect of the linear predictor was assumed to be constant across hospitals. Furthermore, no effect of time was considered in this analysis. This analysis is very similar to random effects meta-analysis of the calibration intercept observed across hospitals, as explored in a companion paper [7].

We considered an extension of Model 3 in which the effect of the linear predictor was allowed to vary randomly across hospitals (Model 4). This model incorporated both random intercepts and a random slope. Thus, both the baseline log-odds of death for a reference patient and the effect of the linear predictor were allowed to vary across hospitals. This analysis is very similar to the random effect meta-analysis of hospital-specific calibration slopes, as explored in the companion paper [7].

Finally, we extended Model 4 to allow the effect of each of the 11 predictors to vary across hospitals, after adjusting for the effect of the linear predictor (Model 5). For this particular set of analyses, we centered the estimated linear predictor around its mean for computational reasons. The interpretation of the hospital-specific effect for the given predictor variable (e.g., age) is as a difference in effect compared to the recalibrated effect as estimated by the previous model. A model of this form has been described previously when examining model validation [10]. Eleven versions of this model were fit, in which the effect of one of the 11 predictors was allowed to vary across hospitals, while the overall effects of the predictors were only allowed to vary according to a calibration slope across hospitals. So, there was essentially a random overall factor for the remaining predictors while we focused on the effect of one specific predictor at a time. We also considered a variant of Model 5, where the effect of the remaining predictors was fixed as in Model 2, which showed similar results. For computational reasons, we were unable to fit a full random coefficients model in which the baseline risk (intercept) and all 11 predictive effects (regression coefficients) varied simultaneously across hospitals. In settings in which *P* values were obtained for a set of statistical tests, we noted the *P* value as larger than the smallest value (“>”).

### Exploring temporal heterogeneity

We explored heterogeneity in baseline risk across time and between hospitals using a random intercept model that incorporated a fixed effect denoting temporal period and a fixed effect for the linear predictor estimated previously (Model 6). In this model, the intercept was allowed to vary across hospitals. Thus, this model allows the baseline risk of 1-year mortality to vary randomly across hospitals as well as systematically between the two time periods.

We then considered temporal variation in the overall predictor effect by extending Model 6 to include an interaction between temporal period and the linear predictor (Model 7). This model allowed the effect of the linear predictor to differ between the two time periods.

In order to examine whether the effect of individual predictors varied temporally, we considered a further extension of the above model, replacing the linear predictor by the 11 covariates in the EFFECT-HF model (Model 8). The resultant model had 12 main effects (one for the temporal period and 11 for the individual predictors) and 11 interactions (interactions between the temporal period and each of the predictors). Thus, the effect of each of the 11 covariates was allowed to differ between the two time periods.

### Simultaneous exploration of geographic and temporal heterogeneity of predictor effects

As an extension to Model 7, we fit a random effects logistic regression model to explore simultaneously geographic and temporal variation in estimated overall predictor effects (Model 9). This model included a random intercept that varied across hospitals, an effect due to the linear predictor that varied across hospitals, a temporal effect that varied across hospitals, and an interaction between these two effects that varied across hospitals. This model permits (i) the effect of the linear predictor to vary between hospitals; (ii) the effect of temporal period to vary across hospitals; and (iii) the effect of temporal period on the predictor effects to vary across hospitals. For computational reasons, we did not attempt to fit a full random coefficients model with interaction by time, in which the baseline risk (intercept) and all 11 predictive effects (regression coefficients) could vary simultaneously across hospitals and across time.

## Results

### Geographic heterogeneity

The regression coefficients were very similar for Model 1 (fixed effects model that ignored geographic and temporal variation) and Model 2 (random intercept model with hospital-specific random intercepts) (Table 1). For a given covariate, the regression coefficient from the second model differed by less than 0.9% from the corresponding coefficient from the first model.

**Table 1:** Estimated odds ratios from fixed effects and random intercept model

Variable	Model 1—fixed effects model		Model 2—random intercept model	
	Odds ratio	95% confidence interval	Odds ratio	95% confidence interval
Age (per year increase)	1.042	(1.038, 1.047)	1.043	(1.039, 1.047)
Systolic blood pressure (per mmHg)	0.987	(0.985, 0.988)	0.987	(0.985, 0.988)
Respiratory rate (per breath)	1.026	(1.019, 1.032)	1.025	(1.019, 1.031)
Serum urea nitrogen	1.105	(1.096, 1.114)	1.106	(1.097, 1.115)
Low-sodium serum concentration (<136 mEq/L)	1.365	(1.249, 1.493)	1.364	(1.246, 1.493)
Low serum hemoglobin (<10.0 g/dL)	1.181	(1.057, 1.319)	1.172	(1.049, 1.310)
Cancer	1.668	(1.492, 1.864)	1.682	(1.504, 1.882)
Chronic obstructive pulmonary disease	1.331	(1.221, 1.450)	1.329	(1.219, 1.450)
Cerebrovascular disease	1.328	(1.207, 1.461)	1.326	(1.204, 1.460)
Hepatic cirrhosis	1.91	(1.253, 2.910)	1.914	(1.253, 2.924)
Dementia	2.124	(1.877, 2.402)	2.136	(1.887, 2.419)

When using Model 2, the hospital-specific random intercepts were estimated to have the following distribution: *N*(*μ* = − 1.25, *σ* = 0.18), with the variance being statistically significantly different from zero (*P* < 0.0001). From the above distribution, the hospital-specific 1-year mortality rates for a reference patient (i.e., one whose standardized covariates were all equal to zero) would lie between 0.167 and 0.292 for 95% of hospitals. The median odds ratio (MOR) (computed using the formula $\mathrm{MOR}= \exp \left(\sqrt{2\times {\sigma}^2}\times 0.6745\right)$, where *σ* ^2^ is the random effects variance estimated above) was equal to 1.19 [11]. Thus, in comparing the odds of death for an individual at a hospital with a higher risk of death with the odds of death for a similar individual at a hospital with a lower risk of death, the median odds ratio over all possible pair-wise comparison of hospitals was 1.19.

The random intercept model in which the intercept varied across hospitals while the effect of the linear predictor was fixed (Model 3) had the following estimated distribution for the random intercepts: *N*(*μ* = 0, *σ* = 0.18), mirroring the same magnitude of between-hospital variation that was observed above. As expected, the estimated regression coefficient for the linear predictor was close to 1 (1.01).

The random coefficient model in which both the intercept and the effect of the linear predictor were allowed to vary across hospitals (Model 4) was found to provide a marginally statistically significant improvement in fit compared to the prior model in which only the intercept varied across hospitals (likelihood ratio test: *χ* ^2^ = 6.08 (*df* = 2), *P* = 0.0478). Assuming a normal distribution for the random effects, the effect of the linear predictor on mortality lies between 0.76 and 1.26 for 95% of hospitals.

Finally, we considered the set of 11 random coefficient models in which the intercept, the linear predictor, and the effect of one of the covariates were allowed to vary across hospitals (Model 5). For each model, we tested whether the three variance-covariance terms associated with the covariate were simultaneously equal to zero. For two of the models (effect of serum urea and the effect of cancer), the test could not be conducted for computational reasons. Of the remaining nine variables, only the presence of low sodium was found to have an effect that varied across hospitals (likelihood ratio test: *χ* ^2^ = 11.43 (*df* = 3), *P* = 0.0096). However, for the remaining eight comparisons, the simpler random coefficients model, in which the effect of the covariate was fixed across hospitals, was found to be acceptable (*P* > 0.24 for the other eight tests). For the model in which the effect of low sodium was allowed to vary, the hospital-specific regression coefficients for the effect of low sodium were found to come from the following distribution: N(0.01, *σ* = 0.29). Thus, the hospital-specific odds ratios for low sodium lay between 0.57 and 1.80 for 95% of hospitals. For all 11 models, the average effect of the covariate, after adjusting for the linear predictor, was not statistically significant (regression-based test of fixed effect: *P* > 0.69).

We conclude that there was no strong evidence for heterogeneity in predictor effects, while baseline risks substantially varied between hospitals.

### Temporal heterogeneity

The crude (unadjusted) probability of death within 1 year was 0.325 and 0.315 in the first and second phase, respectively. The regression coefficient for the main effect of temporal period was −0.111 (odds ratio 0.89, 95% CI = (0.83, 0.97), *P* = 0.0050), in the random intercept logistic model in which the outcome was regressed on the linear predictor and an indicator variable denoting temporal period (Model 6). Thus, the adjusted odds of death were 11% lower in the second phase than those in the first phase of the study, providing evidence of temporal improvement in the risk of 1-year mortality.

The interaction between the linear predictor and the temporal period indicator was not statistically significant (interaction point estimate = −0.007, 95% CI = (−0.094, 0.081), *P* = 0.883, Model 7). Thus, there was no evidence that the effect of the linear predictor differed between the two time periods.

When the above analysis was repeated with the linear predictor replaced by the 11 individual predictor variables (Model 8), comparable results were observed with one exception: while the effects of 10 of the 11 predictor variables did not change over time (test of fixed effect from the fitted regression model: *P* > 0.067 for these 10 tests), the effect of cirrhosis differed between the two time periods (odds ratio 2.98 in phase 1 vs 1.12 in phase 2, *P* value for interaction = 0.027).

### Simultaneous exploration of geographic and temporal stability

In Model 9, we found no evidence that, on average, the effect of the linear predictor differed between the two time periods (interaction term = −0.0008, 95% CI = (−0.1000, 0.0984), *P* = 0.99). Furthermore, a test of the hypothesis that the four variance-covariance terms associated with the interaction was not statistically significant (likelihood ratio test: *χ* ^2^ = 1.20 (*df* = 4), *P* = 0.88). Consequently, we refit Model 9 after eliminating the interaction term (this removed one fixed effect—the interaction term and four variance-covariance terms—those terms involving the correlation between the random effect for the interaction and the random effects for the other three random effects). In this reduced model, a test of the hypothesis that the three variance-covariance terms associated with either the temporal effect were simultaneously equal to zero was not statistically significant (likelihood ratio test: *χ* ^2^ = 6.25 (*df* = 3), *P* = 0.10). Consequently, the effect of time did not vary across hospitals. However, a test of the hypothesis that the five variance-covariance terms involving the linear predictor or the temporal effect were all simultaneously equal to zero was statistically significant (likelihood ratio test: *χ* ^2^ = 12.33 (*df* = 5), *P* = 0.03). Thus, there was evidence that the effect of the linear predictor varied across hospitals, even after accounting for the temporal effect. A limitation of these analyses is that it is unclear what the statistical power is for testing that the three variance-covariance terms were simultaneously equal to zero. Even in our relatively large dataset, the test may have low statistical power. A second limitation is that even when the test is statistically significant, as in the latter case, it is unclear whether there is an appropriate measure of effect size (aside from reporting the individual variance-covariance terms).

## Discussion

Clinical prediction models are intended for widespread application in health care, including use in subjects different from those in whom the model was developed. An emerging aspect of assessing model transportability is assessing the heterogeneity of estimated covariate effects across time and across centers. We illustrated the use of random effects regression models for examining this temporal and geographic heterogeneity in baseline risk and in the estimated predictor effects.

Using data on patients hospitalized with heart failure, we found that temporal and geographic variation in predictor effects was minimal. In contrast, the probability of the occurrence of the outcome (“baseline risk”) was found to vary substantially between centers and between time periods. These analyses complement classical methods for assessing model validity reported in a companion article [7]. In this companion article, we also found that the EFFECT-HF mortality prediction model displayed good temporal and geographic transportability in terms of discrimination and calibration slope when assessed using an internal-external validation approach. The calibration intercept varied in a similar way to the random effect estimate in the current analyses (Models 3 and 4).

An advantage to the methods illustrated in this paper is that they allow all subjects to be included in model development, without the necessity of withholding some subjects for model validation. This increases model stability, due the larger number of subjects used for model development. In developing prediction models, the desire is for a model that is valid everywhere. The examination of geographic and temporal variation in predictor effects permits an exploration of whether this holds true for a given model. Many *P* values were reported to test null hypotheses related to stable effects of baseline risk and predictors. Alternatively, we could qualitatively examine model performance measures, specifically calibration of predictions in time and place. While predictor effects can be anticipated to be fixed geographically or temporally in many settings, this may not be universally true. Certain centers may have more experience and expertise in treating more acutely ill patients, which could diminish the predictive effect of covariates at those hospitals. A more frequent occurrence is that in which the baseline line risk of the outcome varies geographically or temporally. This can result in the developed model displaying lack of calibration when applied in different settings. An example is the validation of the Framingham model to predict cardiovascular disease, in which the baseline risk was found to vary between ethnically diverse populations [12]. Similar systematic miscalibration was observed for the prediction of indolent prostate cancer in a clinical versus a screening setting [13].

A limitation to relying solely on the methods described in the current paper is the lack of a global measure of model performance such as the *c*-statistic, the Brier Score, and the generalized *R* ^2^ statistic. Such measures can be used for a comparison of the relative performance of competing prediction models. Accordingly, assessing variation in predictor effects can best be seen as complementary, providing important information about the geographic and temporal portability of a particular prediction model.

**Fig. 1:**
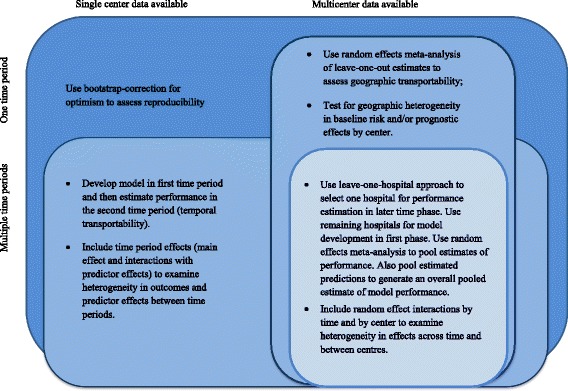
Recommendations for validating clinical prediction models

Furthermore, we were unable to fit all of the desired models. We attempted to fit a random coefficients logistic regression model in which the intercept and the effects of all 11 covariates were allowed to vary across hospitals. In Fig. 1, we summarize graphically some recommendations for assessing geographic and temporal portability of clinical prediction models, based on our analyses in this paper and in a companion study [7] (note that this figure is an expansion of that provided in our earlier article in which we did not consider temporal and geographic stability of predictor effects). We provide recommendations for scenarios ranging from the simple, consisting of data from a single center at a single time period, to the complex, consisting of data from multiple centers or hospitals at multiple time periods. We note that estimates of heterogeneity in baseline risk in the current paper match well with the heterogeneity in calibration intercept in a random effects meta-analysis in the companion paper. Similarly, the limited heterogeneity in effect of the linear predictor was noted here (model 4) and in the meta-analysis of the calibration slope in the companion paper. The extension in the current paper to heterogeneity in effect of individual predictors overall (model 5) or by time (model 8) is not possible in the classical approach to model validation, although this heterogeneity should be reflected in heterogeneity in the *c*-statistic.

We have described a comprehensive suite of analyses that permit one to examine geographic and temporal stability of baseline risk and estimated covariate effects. However, in some settings, analysts may not be able to apply all of these methods. For instance, if data were only available from one time period, then one would not be able to examine temporal stability. In such a setting, one would be limited to examining geographic stability of baseline risk and estimated covariate effects. Thus, the described set of analyses may need to be modified to accommodate the nature of the available data.

There are different contexts in which the methods described in this paper may be applied in practice. Damen et al. conducted a systematic review of prediction models for cardiovascular disease in the general population [14]. They concluded that there was a surfeit of models for predicting incident cardiovascular disease. Instead of developing new prediction models, we agree that greater energy should be expended on externally validating existing models and on conducting head-to-head comparisons of existing models. When considering two models whose performance on global performance measures (e.g., the *c*-statistic) are comparable, one would prefer the model that demonstrated greater temporal and geographic stability. Similarly, when developing a new prediction model, one would prefer to retain those variables for which there was temporal and geographic stability of their effects, as this would increase the likelihood that the model would be subsequently undergo successful external validation.

## Conclusions

The estimation-based methods described in the current study complement classical methods for model validation. These methods allow one to directly examine geographic and temporal heterogeneity in baseline risk as well as variation in predictor effects.

## Appendix

**Table:** Mathematical description of statistical models used for studying model variation

Model	Model description	Description
Ignoring temporal and geographic variation		
Model 1	logit(*p*_*ij*_) = *α*_0_ + *βX*_*ij*_ where *p*_*ij*_ denotes the probability of the outcome for the *i*th patient at the *i*th hospital. From this model, we extracted the linear predictor (LP_*ij*_)	Fixed effects model, ignoring temporal and geographic heterogeneity
Models accounting for geographic heterogeneity		
Model 2	logit(*p*_*ij*_) = *α*_0*j*_ + *βX*_*ij*_ where the hospital-specific random effects *α*_0*j*_ ~ *N*(*α*_0_, *σ* ^2^)	Random intercept model, allowing for variation in baseline risk, but assuming common prognostic effects
Model 3	logit(*p*_*ij*_) = *α*_0*j*_ + *α*_1_LP_*ij*_ where the hospital-specific random effects *α*_0*j*_ ~ *N*(*α*_0_, *σ* ^2^)	Rank 1 model, allowing for common effect of the linear predictor
Model 4	logit(*p*_*ij*_) = *α*_0*j*_ + *α*_1*j*_LP_*ij*_ where $$ \left(\begin{array}{l}{\alpha}_{0 j}\\ {}{\alpha}_{1 j}\end{array}\right)\sim \mathrm{M}\mathrm{V}\mathrm{N}\left(\left(\begin{array}{l}{\alpha}_0\\ {}{\alpha}_1\end{array}\right),\left(\begin{array}{l}{\sigma}_1^2\kern0.5em {\sigma}_{12}\\ {}{\sigma}_{12}\kern0.5em {\sigma}_2^2\end{array}\right)\right) $$ The distribution of the random effects was estimated to be $$ \left(\begin{array}{l}{\alpha}_{0 j}\\ {}{\alpha}_{1 j}\end{array}\right)\sim \mathrm{M}\mathrm{V}\mathrm{N}\left(\left(\begin{array}{l}0.005\\ {}1.008\end{array}\right),\left(\begin{array}{l}0.0444\kern1em 0.0139\\ {}0.0139\kern1em 0.0162\end{array}\right)\right) $$	Rank 1 model, allowing for heterogeneity in the effect of the linear predictor
Model 5	logit(*p*_*ij*_) = *α*_0*j*_ + *α*_1*j*_LP_*ij*_ + *α*_2*j*_ *X*_1*ij*_ where $$ \left(\begin{array}{l}{\alpha}_{0 j}\\ {}{\alpha}_{1 j}\\ {}{\alpha}_{2 j}\end{array}\right)\sim \mathrm{M}\mathrm{V}\mathrm{N}\left(\left(\begin{array}{l}{\alpha}_0\\ {}{\alpha}_1\\ {}{\alpha}_2\end{array}\right),\left(\begin{array}{l}{\sigma}_1^2\kern0.5em {\sigma}_{12}\kern0.5em {\sigma}_{13}\\ {}{\sigma}_{12}\kern0.5em {\sigma}_2^2\kern0.5em {\sigma}_{23}\\ {}{\sigma}_{13}\kern0.5em {\sigma}_{23}\kern0.5em {\sigma}_3^2\end{array}\right)\right) $$ and *X*_1*ij*_ denote an individual predictor (e.g., age)	Fully stratified model, allowing for differential prognostic effects (one model per covariate)
Models accounting for temporal heterogeneity		
Model 6	logit(*p*_*ij*_) = *α*_0*j*_ + *α*_1_ *T*_*ij*_ + *α*_2_LP_*ij*_ where the hospital-specific random effects *α*_0*j*_ ~ *N*(*α*_0_, *σ* ^2^) and the fixed effect for LP_*ij*_ are defined as in Model 3, and *T*_*ij*_ denotes the temporal period (*T* = 0 for phase 1 vs *T* = 1 for phase 2)	Random intercept model with a fixed main effect for phase 2 vs phase 1
Model 7	logit(*p*_*i*_) = *α*_0*j*_ + *α*_1_ *T*_*ij*_ + *α*_2_LP_*ij*_ + *α*_3_ *T*_*ij*_ × LP_*ij*_	Random intercept model with a fixed interaction effect for phase 2 vs phase 1. The prognostic effect differs between time periods
Model 8	logit(*p*_*i*_) = *α*_0*j*_ + *α*_1_ *X*_*ij*_ + *α*_2_ *T*_*ij*_ + *α*_3_ *T*_*ij*_ × *X*_*ij*_	Random intercept model that allowed effect of each predictor to vary between time periods
Simultaneous exploration of geographic and temporal heterogeneity of predictor effects		
Model 9	logit(*p*_*ij*_) = *α*_0*j*_ + *α*_1*j*_LP_*ij*_ + *α*_2*j*_ *T*_*ij*_ + *α*_3*j*_ *T*_*ij*_ × LP_*ij*_where $$ \left(\begin{array}{l}{\alpha}_{0 j}\\ {}{\alpha}_{1 j}\\ {}{\alpha}_{2 j}\\ {}{\alpha}_{3 j}\end{array}\right)\sim \mathrm{M}\mathrm{V}\mathrm{N}\left(\left(\begin{array}{l}{\alpha}_0\\ {}{\alpha}_1\\ {}{\alpha}_2\\ {}{\alpha}_3\end{array}\right),\left(\begin{array}{l}{\sigma}_1^2\kern0.5em {\sigma}_{12}\kern0.5em {\sigma}_{13}\kern0.5em {\sigma}_{14}\\ {}{\sigma}_{12}\kern0.5em {\sigma}_2^2\kern0.5em {\sigma}_{23}\kern0.5em {\sigma}_{24}\\ {}{\sigma}_{13}\kern0.5em {\sigma}_{23}\kern0.5em {\sigma}_3^2\kern0.5em {\sigma}_{34}\\ {}{\sigma}_{14}\kern0.5em {\sigma}_{24}\kern0.5em {\sigma}_{34}\kern0.5em {\sigma}_4^2\end{array}\right)\right) $$	The effect of the linear predictor varies between hospitals; the effect of temporal period varies across hospitals; and the effect of temporal period on the predictor effects varies across hospitals
